# Profiling the Gut Microbiota in Obese Children with Formula Feeding in Early Life and Selecting Strains against Obesity

**DOI:** 10.3390/foods13091379

**Published:** 2024-04-30

**Authors:** Cong Liang, Lan-Wei Zhang

**Affiliations:** 1 College of Safety and Environmental Engineering, Shandong University of Science and Technology, Qingdao 266590, China; liangcong@sdust.edu.cn; 2 School of Chemistry and Chemical Engineering, Harbin Institute of Technology, Harbin 150010, China; 3 College of Food Science and Engineering, Ocean University of China, Qingdao 266003, China

**Keywords:** formula-fed children, obesity, probiotic, gut microbiota, CCK

## Abstract

Formula feeding, obesity and the gut microbiota are closely related. The present investigation explored the profiles of the intestinal microbiota in obese children over 5 years old with formula feeding in early life. We identified functional bacteria with anti-obesity potential through in vitro and in vivo experiments, elucidating their mechanisms. The results indicated that, in the group of children over 5 years old who were fed formula in early life, obese children exhibited distinct gut microbiota, which were characterized by diminished species diversity and reduced *Bifidobacterium* levels compared to normal-weight children. As a result, *Lactobacillus acidophilus* H-68 (H-68) was isolated from the feces of the N-FF group and recognized as a promising candidate. H-68 demonstrated the ability to stimulate cholecystokinin (CCK) secretion in STC-1 cells and produce bile salt hydrolase. In vivo, H-68 promoted CCK secretion, suppressing food intake, and regulated bile acid enterohepatic circulation, leading to increased deoxycholic acid and lithocholic acid levels in the ileum and liver. This regulation effectively inhibited the diet-induced body weight and body fat gain, along with the liver fat deposition. In conclusion, H-68 was recognized for its prospective anti-obesity impact, signifying an auspicious pathway for forthcoming interventions targeted at averting pediatric obesity in formula-fed children.

## 1. Introduction

In light of the pivotal time frame for shaping the configuration of the human gut microbiota prior to the age of three, the early feeding practices of infants can exert influence on its distinctive characteristics [1]. Currently, many children’s milk powders are supplemented with probiotics to mitigate growth issues associated with early formula feeding, with the most prevalent being *Bifidobacterium animalis* BB12 [2,3,4]. This strain stands out as the sole *Bifidobacterium* with a complete gene sequence. In the realm of pediatrics, BB-12 has demonstrated efficacy in treating acute and chronic diarrhea, treating antibiotic-related diarrhea, and providing auxiliary therapeutic effects for functional constipation in children [5,6,7]. Nevertheless, in addition to digestive problems linked to early-life constipation, formula feeding has been established to be closely associated with later obesity [8]. This underscores the need for further research and development of functional probiotics tailored for populations relying on formula feeding. Prior to this, investigating the structural characteristics of the gut microbiota in obese children fed with milk powder can serve as a catalyst for advancing our understanding.

Indeed, numerous reports have highlighted the efficacy of probiotics in combating obesity, with the prevalent strains including *Lactobacillus*, *Bifidobacterium*, *Akkermansia*, and others. For instance, Shan et al. [9] recently identified a *Lactobacillus reuteri* strain with anti-obesity effects, elucidating its function through the metabolite Indole-3 *Carboxaldide* in obese mice. Chen et al. [10] showcased that *L. plantarum* HF02 effectively restrained fat accrual and mitigated disruptions to the gut microbiota of diet-induced obese mice. Similarly, Ban et al. [11] uncovered that *Bifidobacterium* lactis IDCC 4301 regulated lipid metabolism against obesity in a mice model. Regarding *Akkermansia*, Gao et al. [12] provided evidence supporting the anti-obesity effects of *Akkermansia* muciniphila-directed polyphenol chlorogenic acid intervention in mice. What is more, *Akkermansia* is a potential anti-obesity functional probiotic that has only been used in clinical trials in adults and animals, but it is not currently used in infant formulae. In summary, different bacterial strains exhibit diverse mechanisms and effects in combating obesity, with variations in their applicability.

Currently, the primary strategies for anti-obesity involve reducing food intake, increasing physical activity and regulating lipid metabolism processes. Researchers have indicated the intricate interconnections within the microbiota–gut–brain axis, encompassing immune, endocrine, systemic, and neural pathways [13,14]. The gut microbiota assumes a pivotal role in modulating feeding behavior by impacting the secretion of appetite-regulating hormones from intestinal endocrine cells. Furthermore, the microbes in our intestines and microorganism metabolites actively participate in intervening in the lipid metabolism processes within the intestine and liver [13,15]. In this study, we recruited a cohort of children with formula feeding in early life with obesity or normal weight and conducted a differential analysis of the gut microbiota structure. Furthermore, we used in vitro and in vivo experiments to screen the functional bacteria with anti-obesity potential by referring to the results of the difference in the intestinal flora.

## 2. Materials and Methods

### 2.1. Children’s Information

The fecal collection protocol for obese children was approved by the ethics committee of Harbin Children’s Hospital, and the ethics number is NO. HRYLL201606. Meanwhile, written informed consent and assent were obtained from a parent or guardian and the child, respectively, to participate in the current study. This study collected children in only Harbin, China, which decreased the error caused by other influencing factors, such as geography and dietary habits, in the research results. In this study, formula-fed children were defined as those who had been exclusively formula fed (not exposed to breast milk) during early life and had been fed for more than 1 year. All the participating children exhibited no congenital diseases, had not encountered gastrointestinal issues like diarrhea, and refrained from antibiotic usage within three months. The classification of the children into obesity adhered to the body mass index (BMI) guidelines for individuals aged 5–19 years, as stipulated by the World Health Organization. This investigation ultimately enrolled 45 children exclusively subjected to formula feeding in their early life, comprising 17 classified as obese children (designated as the OB-FF group) and 28 categorized as normal-weight children (designated as the N-FF group). The basic information of the children is shown in Table 1.

### 2.2. Collection of Fresh Fecal Samples and Processing

The collection of clean feces from the children required the help of the parents. We provided the parents with sterile fecal containers containing 80% glycerol and 20% MRS liquid culture medium for storing feces. The gathered fecal samples were preserved at a temperature of −80 °C.

### 2.3. High-Throughput 16SrDNA Gene Sequencing and Data Processing

The high-throughput 16S rDNA gene sequence and data processing were conducted by BGI Co. The experimental sequence comprised DNA extraction, determining the purity and concentration of the DNA, PCR amplification targeting the 16S rDNA V4 hypervariable region, evaluation of the DNA fragment integrity, library construction, and high-throughput sequencing (Illumina Miseq platform). The quantity and quality of the extracted DNA were detected using the fluorescence quantitative method (Qubit BR ds DNA Assay Kit, Thermo Fisher Scientific, Waltham, MA, USA) and agarose gel electrophoresis, respectively. The detailed decomposition of the experimental sequence was consistent with Gao et al. [16]. The primer sequences used in PCR amplification were 515F: GTGCCAGMGCCGCGGTAA and 806R: GGAC-TACHVGGGTWTCTAAT, respectively.

### 2.4. Isolation and Identification of Bacteria Strains

The isolation of presumed *Bifidobacterium* adhered to the procedure delineated by Kneifel et al. [17], whereas the isolation of presumed *Lactobacillus* was executed according to the methodology of McCoy et al. [18]. Approximately 1 g of stool sample was collected in sterile physiological saline and diluted to 10–6 times. Then, 100 μL of diluted stool suspension was, respectively, taken on LBS and MRS-X-gal solid medium, evenly coated, and incubated at 37 °C for 48 h. After the culture, the colonies with round, white (or light yellow) or translucent colonies were selected from the LBS solid medium for microscopic examination. If an elongated and unbranched thallus was observed in the long, short or rod shape, it was suspected to be *Lactobacillus*. Blue colonies were selected from the MRS-X-gal solid medium for microscopic examination. If the cells were observed to be rod-shaped and bifurcated at one or both ends, they were suspected as *Bifidobacterium*. The selected colonies were isolated and purified by the plate scribe method, enriched and cultured, and stored in MRS liquid medium containing 20% glycerol, and stored at −80 °C until use. Species identification through 16S rRNA gene sequencing was carried out in accordance with the approach detailed by Wang et al. [19].

### 2.5. Selection of Strains with the Ability of Stimulating CCK Secretion

The selection of strains for stimulating cholecystokinin (CCK) secretion from STC-1 cells adhered to the procedures expounded by Santos-Hernández et al. [20] and Wei et al. [21]. The STC-1 cells used in this study were purchased from Shanghai Enzyme Research Biotechnology Co., Ltd. (Shanghai, China). The STC-1 cells were cultured in DMEM high-sugar medium (Hyclone, UT, USA) containing 10% fetal bovine serum (Gibco Thermo Fisher Inc., Waltham, MA, USA) and l-glutamine (4 mM) at 37 °C with an atmosphere of 5% CO_2_ and 95% air. For the CCK secretion analysis, the cells were divided into 24-well plates (2 × 10^5^ cells per well), respectively, for 2 h. Mouse ELISA kits (Langton, Shanghai, China) were employed to assess the CCK levels in the supernatant. In summary, optimal conditions were upheld for the co-cultivation of *Lactobacillus* strains and STC-1 cells.

### 2.6. Qualitative Screening of Bile Salt Hydrolase (BSH)-Producing Strains

The BSH production ability of the strains was qualitatively assessed using a plate precipitation method adapted from Dashkevicz et al. [22]. In brief, freshly prepared MRS liquid culture medium was supplemented with 0.3% (*m*/*v*) taurine ursodeoxycholic acid sodium, 0.37 g/L CaCl₂, 0.2% (*m*/*v*) sodium mercaptoacetate, and 1.5% (*m*/*v*) agar. This mixture was sterilized at 121 °C for 15 min. Subsequently, the sterile liquid culture medium was poured into sterile plates containing multiple Oxford cups. After solidification, each Oxford cup was extracted, and 10 μL bacterial solution (10^9^ CFU/mL) of suspected *Lactobacillus* and *Bifidobacterium* strains was introduced into the circular well. The plates were then placed under anaerobic conditions at 37 °C for 48 h. Post-cultivation, the presence of white precipitates around the circular pores indicated the strain’s ability to produce BSH, while the absence of such precipitates indicated otherwise. A blank control was established using MRS solid culture medium without bile salts, and each strain was subjected to three repetitions.

### 2.7. Animals and Experimental Design

*Animals.* Male C57BL/6J mice, numbering 34 and aged 3 weeks, were procured from Beijing Huafukang Biotechnology Co., Ltd. (Beijing, China). They were housed in a specific-pathogen-free (SPF) environment, ate and drank freely, and were maintained in conditions of 22 ± 1.0 °C, 55 ± 5% RH, and 12:12 h of day and night cycle. The experimental protocols in this study were approved by the Welfare Ethics Committee of Laboratory Animals of Harbin Institute of Technology (Approval No. IACUC-2019018). The low-fat diet (D12450B, 10% fat) and high-fat diet (D12492, 60% fat) were procured from Beijing Keao Xieli Feed Co., Ltd. (Beijing, China).

*Evaluation of strain presence in the murine intestine.* The mice received intragastric administration of live-strain cells of *L. acidophilus* H-68, tagged with 5-(and 6)-Carboxyfluorescein diacetate, succinimidyl ester (Solarbo, Beijing, China). The dosage was 200 μL (5 × 10^8^ CFU/mL) *L. acidophilus* H-68 suspension per mouse. Then, the mice were euthanized, and ileum tissue was collected at 6 h, 12 h, 18 h, and 24 h post-administration. The presence of *L. acidophilus* H-68 in the mouse ileum was evaluated using a fluorescence imaging system (IVIS Lumina XRMS Series III, PerkinElmer, Waltham, MA, USA) with an excitation wavelength of 488 nm and an emission wavelength of 508 nm.

*Analysis of L. acidophilus H-68’s anti-obesity effect in vivo.* Thirty mice, after adaptive feeding for one week, were stochastically allocated into three groups as follows: mice fed a low-fat diet (LFD group), mice fed a high-fat diet (HFD group), and mice fed a high-fat diet and 200 μL (5 × 10^8^ CFU/mL) *L. acidophilus* H-68 suspension per mouse every day (H-68 group). The experimental process lasted for 8 weeks. After the feeding, all the mice were sacrificed by cervical dislocation.

### 2.8. Measurement and Histological Analysis

Throughout the study, weekly recordings were made of the body weight and food intake. At the conclusion of the experiments, the mice were sacrificed following a 12 h fasting period, and the serum was extracted and stored at a temperature of −80 °C. Perirenal, epididymal, and inguinal white adipose tissue (commonly abbreviated as pWAT, eWAT, and iWAT), along with the liver, were harvested, cleansed, and weighed. These tissues were also soaked in 10% formalin for the histological analysis, including the slicing, dehydration, and H&E staining processes.

### 2.9. Biochemical Analysis

A fully automated chemical analyzer, a Cobas C 311 (Roche Diagnostics, Mannheim, Germany), was adopted to obtain the serum parameters, including the cholesterol (CHOL), alanine aminotransferase (ALT), high-density lipoprotein (HDL), triglycerides (TG), aspartate aminotransferase (AST), and low-density lipoprotein (LDL). Moreover, the corresponding mouse ELISA kits (Langton, Shanghai, China) were adopted to test the serum levels of tumor necrosis factor-α (TNF-α), lipopolysaccharide (LPS), and fibroblast growth factor 15 (FGF15). Furthermore, the TG and CHOL assay kits (Solarbo, Beijing, China) were adopted to obtain the liver TG and CHOL levels, respectively.

### 2.10. Bile Salt Analysis

The lithocholic acid (LCA) and deoxycholic acid (DCA) contents were determined according to the procedure outlined by Liu et al. [23]. Briefly, 50 mg of ileal content or liver homogenate sample and 500 mL of pure methanol were fully mixed. One magnetic bead was added to the resulting solution, followed by vibration for 240 s at 70 Hz and centrifugation (12,000 rpm, 10 min) at 4 °C. The quantification of the LCA and DCA levels in the supernatant was carried out using an Agilent 6470 Triple Quadrupole MS/MS coupled with an Agilent 1290 Infinity UHPLC system (Agilent, Santa Clara, CA, USA). Separation was achieved on an X Select HSS T3 column (2.1 mm × 100 mm, 2.5 μm) at 40 °C and a flow rate of 0.3 mL/min. Water (solvent A, containing 0.1% formic acid aqueous solution and 10 mM ammonium formate) and acetonitrile (solvent B) served as the mobile phase for the gradient elution. The negative ion mode (Gas Flow 9 L/min, Gas Temp 350 °C, Nebulizer 40 Psi, SheathGasHeater 300 °C, VCharging 500 V, SheathGasFlow 11 L/min, Capillary 3500 V) was employed for the electrospray ionization, with nitrogen as the nebulizer. Quantification was accomplished using the multiple reaction monitoring mode.

### 2.11. Real-Time Quantitative PCR

The total RNA of the liver and ileum was extracted using the EasySpin Plus Tissue/Cell RNA Extraction Kit (Aidlab, Beijing, China). The gene expressions were analyzed using the CFX96 real-time PCR detection system (Bio-Rad, Munich, Germany) and the FastKing One Step Reverse Transcription Fluorescent Quantitative Kit (SYBR Green) (Tiangen, China) (Table 2). The PCR primers were supplied by the Beijing Genomics Institute (Beijing, China). *β-actin* served as the housekeeper gene to normalize the target gene levels using the 2^−ΔΔCt^ method.

### 2.12. Statistical Analysis

Data processing of the intestinal microbiota included measures of the α diversity (Shannon’s diversity and Simpson’s diversity), species composition analysis at various levels, and partial least squares discrimination analysis (PLS-DA).

The data were analyzed using SPSS 22.0 (SPSS Inc., Chicago, IL, USA). The differences in the values of the CCK level, body weight, food intake, body fat, blood lipid, inflammatory index, bile acid level, and liver lipid metabolism index were determined with a one-way ANOVA by Tukey’s post hoc tests. The differences in the values of the children’s age, height, body weight, and BMI index were analyzed by unpaired two-tailed *t* test. All the experiments in this study were repeated three times. The outcomes were conveyed as the mean ± standard deviation (SD). A significance threshold of *p* < 0.05 was deemed statistically noteworthy.

## 3. Results

### 3.1. Intestinal Microbial Community of Obese Formula-Fed Children

**Figure 1:**
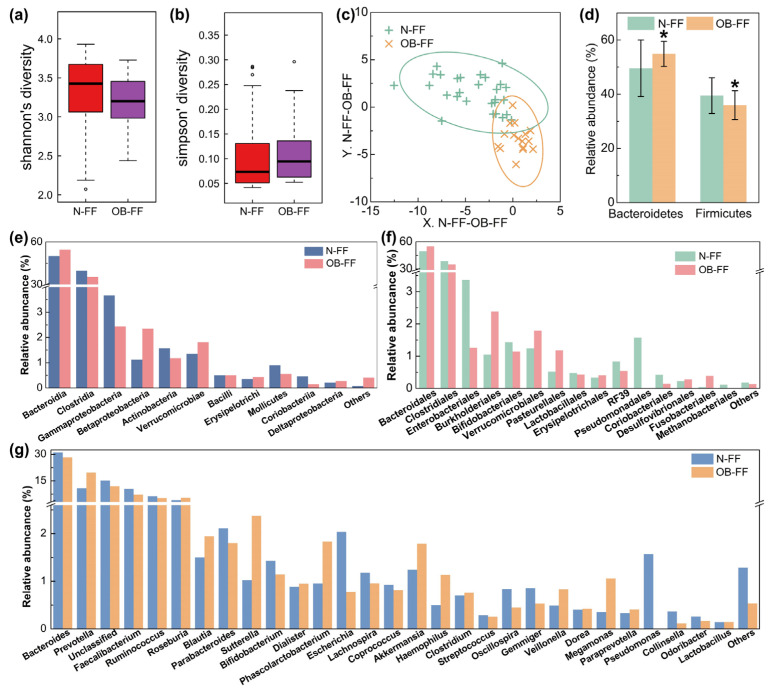
Gut microbiota structure of obese and normal-weight children fed with formula: (**a**) Shannon’s diversity, (**b**) Simpson’s diversity, (**c**) PLS-DA results, (**d**) phylum level, (**e**) order level, (**f**) class level, and (**g**) genus level. N-FF: normal-weight formula-fed children; OB-FF: obese formula-fed children. * *p*  <  0.05.

The fecal samples from the children underwent analysis for the microbial structure. The disparity in bacterial diversity, as indicated by the Shannon’s and Simpson’s indices, was computed. Notably, a decreased value of Shannon‘s and increased Simpson’s were observed, even if there was no significant difference (Figure 1a,b). PLS-DA revealed a discernible clustering of microbiota composition for each group, and the samples from the same group were clustered (Figure 1c), which suggested a difference in the gut microbiota composition between the N-FF and OB-FF groups. The examination of the relative abundance of the predominant taxa identified via sequencing was conducted to characterize specific alterations in the gut microbiota. As shown in Figure 1d, the relative abundances of the two dominant phylum in the intestine, Firmicutes and Bacteroidetes, were analyzed. Compared with the N-FF group, the Firmicutes in the gut microbiota of the OB-FF group significantly reduced, while the Bacteroidetes prominently increased (Figure 1d, *p* < 0.05). Additionally, a comprehensive depiction of the composition of the intestinal bacteria was provided at the class, order, and genus levels (Figure 1e–g). Notably, attention was drawn to changes in the genera *Lactobacillus*, *Bifidobacterium*, and *Akkermansia*, as they are recognized to be associated with obesity [24]. As shown in Figure 1g, only *Bifidobacterium* was reduced in obese children with formula feeding in early life compared to normal-weight individuals, even if there was no significant difference. Conversely, the *Akkermansia* showed enrichment in the obese subjects, even if there was no significant difference (Figure 1g). The abundance of *Lactobacillus* showed no significant difference between the OB-FF and N-FF groups (Figure 1g).

### 3.2. Screening Strains with Potential Anti-Obesity Function In Vitro

**Figure 2:**
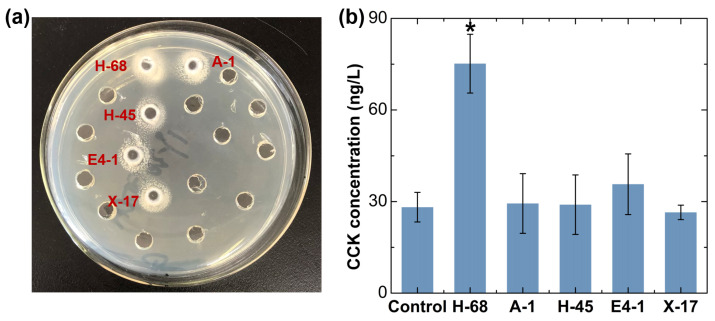
In vitro screening results of potential anti-obesity functional bacteria. (**a**) Screening of strains producing BSH. (**b**) Screening of strains stimulating CCK secretion in STC-1 cells. * *p*  <  0.05.

*Bifidobacterium* was considered as the candidate for the strain with an anti-obesity effect, as it was reduced in obese individuals, even if there was no significant difference (Figure 1g). Considering that *Lactobacillus* have been extensively reported in the field of probiotics against obesity, even though there was no obvious difference in the abundance of *Lactobacillus* between the two groups of samples in our study, we still considered *Lactobacillus* deserved to be a candidate for an anti-obesity functional bacteria strain. Using the selective culture medium, a total of 30 suspected strains of *Lactobacillus* and 28 suspected strains of *Bifidobacterium* were obtained from the feces of children in the N-FF group. Subsequently, we performed a qualitative analysis of the bile salt hydrolase (BSH)-producing ability of these 58 strains, and 5 positive strains were selected for further investigation (Figure 2a). Moreover, we assessed the ability of these 5 strains to stimulate cholecystokinin (CCK) secretion in STC-1 cells, identifying the H-68 strain as having an excellent capability (Figure 2b, *p* < 0.05). The bacterial identification results confirmed that H-68 belongs to the *Lactobacillus acidophilus* strain and that it came from the feces of normal-weight children.

### 3.3. L. acidophilus H-68 Suppressed Food Intake and Obesity in HFD-Fed Mice

**Figure 3:**
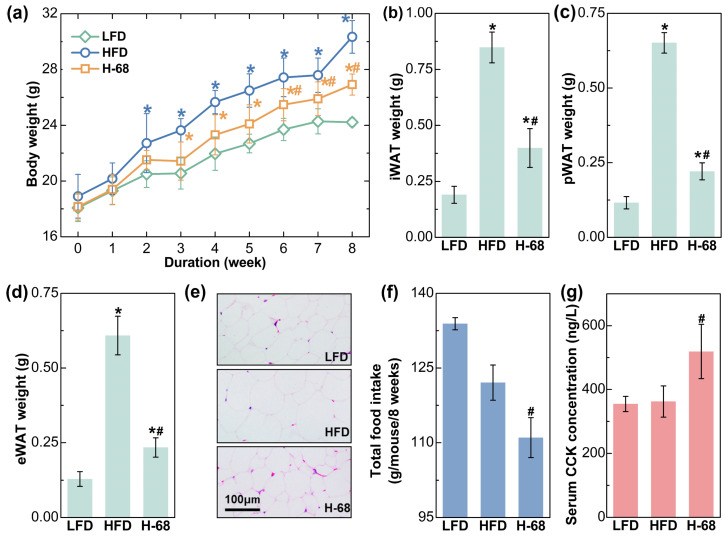
The effects of *L. acidophilus* H-68 on the body weight, body fat and food intake in HFD-fed mice: (**a**) body weight, (**b**) iWAT weight, (**c**) pWAT weight, (**d**) eWAT weight, (**e**) random eWAT sections, (**f**) food intake, and (**g**) serum CCK level. * *p* < 0.05, versus LFD group, # *p* < 0.05, versus HFD group.

As shown in Figure 3a, HFD feeding treatment for 8 weeks led to significant increases in body weight, while *L. acidophilus* H-68 supplementation partially prevented the gain in body weight (*p* < 0.05). Additionally, the weight of the three main types of white adipose tissues of the mice were then measured, and *L. acidophilus* H-68 supplementation effectively prevented the accumulation of adipose tissue (Figure 3b–d, *p* < 0.05), aligning with the observations regarding body weight. The size of the adipocytes could more intuitively reflect the differences in lipid accumulation in the adipocytes. The adipocyte size within the WAT of mice treated with *L. acidophilus* H-68 was smaller than in the HFD group (Figure 3e). Furthermore, *L. acidophilus* H-68 significantly elevated the serum cholecystokinin (CCK) levels and decreased the food intake of mice fed a high-fat diet (Figure 3f,g, *p* < 0.05). These findings indicate that the administration of *L. acidophilus* H-68 suppressed the food intake and resulted in an effective inhibition of HFD-induced obesity development.

### 3.4. L. acidophilus H-68 Inhibited Disorder of Related Indicators of Serum Lipid Metabolism, Liver Injury, and Inflammatory Response in HFD-Fed Mice

**Figure 4:**
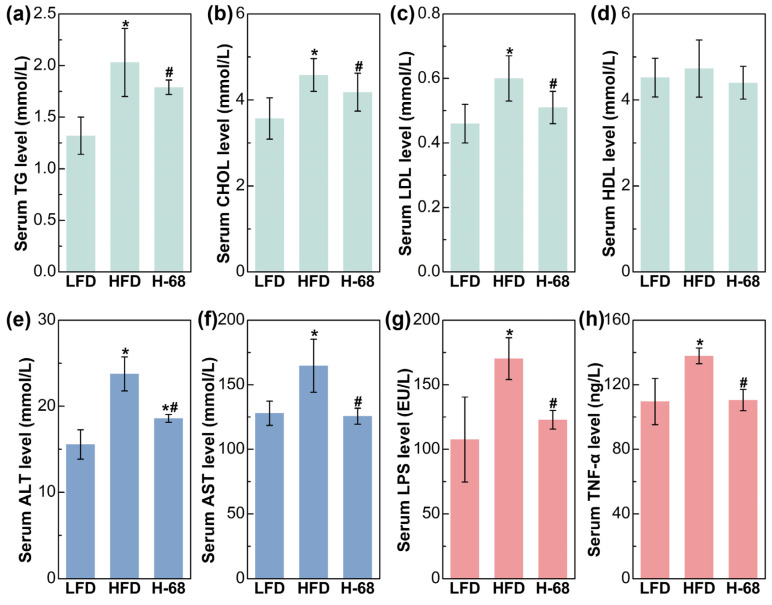
The effects of *L. acidophilus* H-68 on the serum indicators of HFD-fed mice: (**a**) serum triglyceride (TG) level, (**b**) serum cholesterol (CHOL) level, (**c**) serum high-density lipoprotein (HDL) level, (**d**) serum low-density lipoprotein (LDL) level, (**e**) serum alanine aminotransferase (ALT) level, (**f**) serum aspartate aminotransferase (AST) level, (**g**) serum lipopolysaccharide (LPS) level, and (**h**) serum tumor necrosis factor-α (TNF-α) level. * *p* < 0.05, versus LFD group, # *p* < 0.05, versus HFD group.

Regarding the serum lipid metabolism-related indices, mice in the HFD group demonstrated remarkably elevated levels of serum TG, CHOL, and LDL in comparison to the LFD group (Figure 4a, *p* < 0.05). Meanwhile, supplementation with *L. acidophilus* H-68 effectively mitigated the increase in the serum TG, TC, and LDL of mice in the HFD group (Figure 4a, *p* < 0.05). Additionally, there was no obvious difference in the HDL levels between the groups (Figure 4a). Intrahepatic fat accumulation may directly lead to liver injury [25]. As shown in Figure 4b, liver injury in the mice was manifested by increased serum ALT and AST levels. By contrast, *L. acidophilus* H-68 supplementation effectively reduced the ALT and AST levels in the serum (Figure 4b, *p* < 0.05). Regarding the serum inflammatory indices, HFD feeding markedly resulted in elevated serum LPS and TNF-α levels, when compared with the LFD group (Figure 4c, *p* < 0.05). Importantly, *L. acidophilus* H-68 supplementation significantly mitigated the increase in the circulating levels of LPS and TNF-α of the mice in the HFD group (Figure 4c, *p* < 0.05). These findings suggest that *L. acidophilus* H-68 has the potential to effectively alleviate HFD-induced dyslipidemia, liver injury, and inflammatory indices in mice.

### 3.5. L. acidophilus H-68 Regulates Bile Acid Metabolism to Alleviate Liver Fat Deposition in HFD-Fed Mice

**Figure 5:**
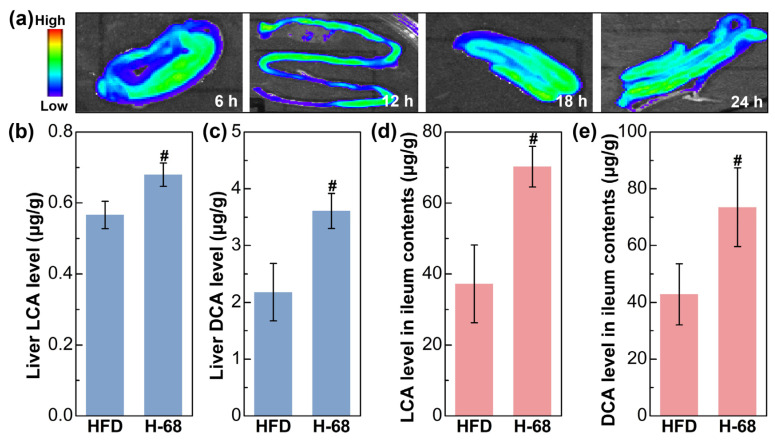
*L. acidophilus* H-68 was planted in the ileum and elevated the deoxycholic acid (DCA) and lithocholic acid (LCA) levels in both the ileum and liver. (**a**) Dynamic fluorescence imaging of *L. acidophilus* H-68 in the ileum. (**b**) The LCA level in the liver. (**c**) The DCA level in the liver. (**d**) The LCA level in the ileum contents. (**e**) The DCA level in the ileum contents. # *p* < 0.05, versus HFD group.

The fluorescence imaging result showed the presence situation of *L. acidophilus* H-68 in the ileum of the mice (Figure 5a). The outcomes depict the ongoing surveillance of the fluorescence intensity within the murine intestines throughout a 24 h time frame, demonstrating the capability of *L. acidophilus* H-68 to establish a presence in the ileum for at least 24 h (Figure 5a). Building upon this observation, *L. acidophilus* H-68 played a role in regulating bile acid metabolism. As shown in Figure 5b, *L. acidophilus* H-68 significantly increased the levels of LCA and DCA in the ileal contents and liver tissues (*p* < 0.05), which was positive correlated to the activation of the *FXR* signaling pathway described below.

**Figure 6:**
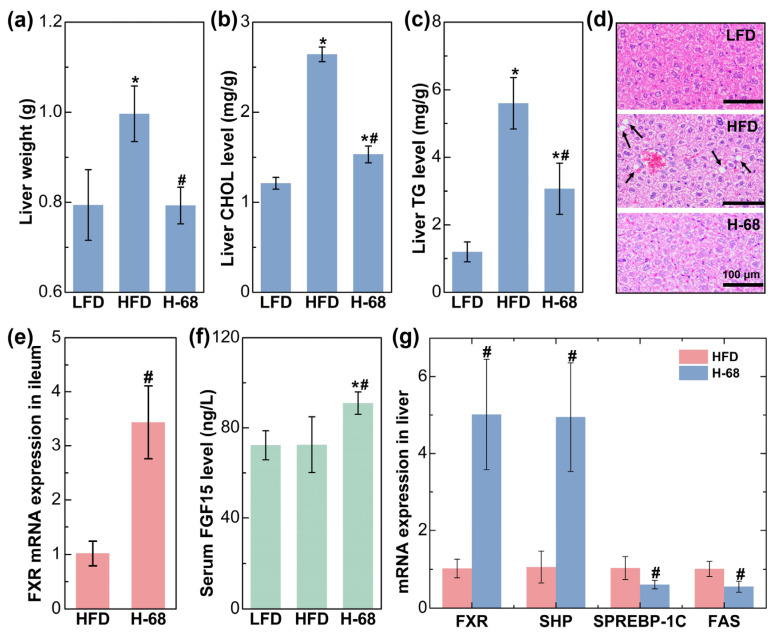
The alleviating effect of *L. acidophilus* H-68 on HFD-induced liver fat deposition depending on activating the *FXR* signaling pathways in mice. (**a**) Liver weight. (**b**) Liver cholesterol (CHOL) concentration. (**c**) Liver triglyceride (TG) concentration. (**d**) Representative liver sections. (**e**) *FXR* mRNA expression in the ileum. (**f**) Serum *FGF15* concentration. (**g**) *FXR*, *SHP*, *SREBP-1C* and *FAS* mRNA expression in liver. * *p* < 0.05, versus LFD group, # *p* < 0.05, versus HFD group.

The liver plays a pivotal role in lipid metabolism, and it actively engages in the synthesis and transportation of endogenous lipids [26]. After an eight-week period of high-fat diet consumption, the mice displayed a significantly elevated liver weight in comparison to those maintained on a low-fat diet. Nevertheless, supplementation with *L. acidophilus* H-68 effectively mitigated this change (Figure 6a, *p* < 0.05). Concurrently, *L. acidophilus* H-68 suppressed the increase in TG and CHOL in the livers of the mice in the HFD group (Figure 6b, c, *p* < 0.05). Macrovesicular steatosis, conspicuous in the livers of the mice in the HFD group but absent in the LFD and H-68 group mice, was prominently observed (Figure 6d). To further elucidate the mechanism by which H-68 alleviates liver lipid deposition, the related indicators of the farnesol X receptor (*FXR*) signaling pathway in the ileum and liver were assessed. The results indicated that H-68 supplementation effectively elevated the mRNA expression of *FXR* in the ileum (Figure 6e, *p* < 0.05), leading to an elevation in the serum fibroblast growth factor 15 (FGF15) levels in the H-68 group mice (Figure 6f, *p* < 0.05). In the liver, supplementation with *L. acidophilus* H-68 significantly enhanced the expression of *FXR* and small heterodimer partner (*SHP*), a downstream gene of *FXR*. This augmentation subsequently inhibited the mRNA expression of sterol regulatory element-binding protein-1c (*SREBP-1C*) and fatty acid synthase (*FAS*), downstream genes of *SHP* and *FXR*, respectively (Figure 6g, *p* < 0.05). In summary, *L. acidophilus* H-68 mitigated liver lipogenesis by activating the pathway of *FXR*/*FGF15* in the ileum and the pathway of *FXR*/*SHP*/*SREBP-1C*/*FAS* in the liver.

## 4. Discussion

Childhood obesity, which is detrimental to physical health, is closely linked to conditions such as precocious puberty in girls, delayed sexual development in boys, and hypertension, posing a threat to mental health as well [27,28,29]. Breastfeeding during the first 4 to 6 months of age was associated with decreased risks of overweight and obesity at the age of 6–7 years [30]. However, the increasing significance of women’s social roles prompts a growing inclination toward formula-feeding. Considering the close association between obesity and the gut microbiota, this study analyzed the difference in the gut microbiota structure between obese and normal-weight individuals in children with early formula feeding, so as to help the acquisition of potential anti-obesity functional bacteria. At the phylum level, the Firmicutes/Bacteroidetes (F/B) ratio of the obese group was decreased compared with the normal-weight group in our study. However, in previous studies, the changes in the F/B ratio of children with obesity varied in different studies, including increasing [31] and remaining unchanged [32]. This may be specific to the group of formula-fed children, which highlights the significance of this study. And it may also be related to factors such as the region and dietary habits, which needs to be demonstrated by researchers in more regions. Furthermore, the obese children displayed diminished diversity and richness in terms of the gut microbiota species, accompanied by a reduced abundance of *Bifidobacterium* (no significant difference). Despite the minimal variation in the relative abundance of *Lactobacillus* in the intestines between the obese and normal-weight children, its well-established recognition in the probiotic domain for its anti-obesity properties led to the consideration of *Lactobacillus* and *Bifidobacterium* as potential strains for anti-obesity interventions in the current paper. And through a series of in vitro and in vivo screening, the potential anti-obesity strain *L. acidophilus* H-68 was obtained.

A part of the anti-obesity potential of *L.* acidophilus H-68 comes from its ability to produce bile salt hydrolase (BSH). BSH, an enzyme secreted by intestinal microorganisms, exhibits the capability to hydrolyze bile acids. Owing to the varying activation potentials of distinct bile acids on the intestinal *FXR* signaling pathway, BSH not only alters the bile acid composition but also ultimately influences the activation of the *FXR* signaling pathway. Given the enterohepatic circulation of bile acids [33], H-68 not only elevated the concentrations of deoxycholic acid (DCA) and lithocholic acid (LCA) in the ileal contents but also increased their content in the liver. Studies have proposed that both LCA and DCA can activate *FXR*, the principal bile acid sensor in the intestine and liver [34]. Activation of *FXR* in ileal enterocytes stimulated the release of FGF15, an enterohepatic hormone capable of reducing liver lipogenesis by activating the *SHP* receptor in the liver [35]. As *SREBP-1C*, the downstream gene of *SHP*, is closely linked to fatty acid biosynthesis and can govern the expression of adipogenesis-related genes, including *FAS*, it regulates fatty acid synthesis [36,37]. In addition, the activation of *FXR* in the liver directly inhibits liver fat synthesis through the *FXR*/*SHP*/*SREBP-1C*/*FAS* signaling pathway. And the suppression of hepatic lipid accumulation mitigated lipotoxicity and liver injury [38], as evidenced by the decreased serum AST and ALT levels in the mice within the H-68 group.

Long-term high-fat diets tend to trigger an inflammatory response, which further promotes the development of obesity [39,40]. It was worth noting that *L. acidophilus* H-68 supplementation significantly mitigated the increase in the serum inflammatory indices of the mice in the HFD group. This may also be related to the increase in the DCA and LCA, which have also been described to stimulate TGR5 (a bile acid-specific G protein-coupled receptor). Therapeutic targeting of TGR5 signaling might be important in restoring normal enterohepatic circulation and controlling inflammation. The activation of TGR5 on enterocytes could increase the synthesis and release of anti-inflammatory cytokines [41]. This suggests that the anti-obesity effect of *L. acidophilus* H-68 may also be related to the activation of the TGR5 pathway by DCA and LCA to alleviate the inflammatory response.

A part of the anti-obesity potential of *L. acidophilus* H-68 comes from its ability to promote cholecystokinin (CCK) secretion. CCK, a hormone secreted by intestinal endocrine cells, possesses the ability to suppress appetite [42]. Elevating the CCK levels is considered an effective strategy in combating obesity [43]. Previous studies have proposed that proteins, carbohydrates, short-chain fatty acids generated by the gut microbiota, and flavonoids can all contribute to stimulating CCK secretion from enteroendocrine cells [44]. However, until the emergence of *L. acidophilus* H-68 in this article, there were few studies on bacterial stimulation of STC-1 cells to induce CCK secretion. In vivo, *L. acidophilus* H-68 has demonstrated the capability to inhibit mouse food intake, closely associated with the elevation of the serum CCK levels. In fact, the level of CCK in obese patients is controversial, as some obese patients show decreased or unchanged CCK levels, while others show increased CCK levels. For obese patients with increased CCK levels, *L. acidophilus* H-68 is not the best choice for adjuvant obesity treatment. Additionally, the suppression of serum inflammatory markers (LPS and TNF-α) was linked to reduced consumption of a high-fat diet, contributing to the effective control of obesity [45,46]. In summary, *L. acidophilus* H-68 alleviated the body weight gain, abdominal fat accumulation, and dyslipidemia. Numerous lactobacillus strains have previously been proven to have anti-obesity effects. For instance, *L. amylovorus* KU4 markedly decreased the body, WAT, and liver weight, thereby improving dyslipidemia and mitigating HFD-induced obesity in mice [47]. Moreover, the synergistic action of lactobacilli demonstrated an obesity prevention effect by notably diminishing the body weight and fat gain, and by improving dyslipidemia [48].

In all candor, this study has merely provided an initial insight into the anti-obesity potential of *L. acidophilus* H-68. Since this study was conducted in mice, the extent of the anti-obesity effect of H-68 in humans needs to be further explored. In the future, we look forward to continuing our clinical studies to evaluate the anti-obesity potential of *Lactobacillus acidophilus* H-68 in children. Moreover, further investigations are imperative for a comprehensive understanding, encompassing: (1) elucidating the mechanism through which *L. acidophilus* H-68 promotes CCK secretion in STC-1 cells, and (2) conducting research on the industrial production and preparation process of *L. acidophilus* H-68. These forthcoming inquiries will contribute to a more nuanced comprehension and potential applications of *L. acidophilus* H-68 in addressing obesity in the specified population. In addition, it is worth mentioning that the sample size of children’s stool in the current study may be insufficient, potentially impacting the reliability of the results. Despite the existence of numerous comparable studies with similar sample sizes [31,49,50,51], we strongly advocate future researchers to undertake investigations with larger sample sizes to enhance and augment the existing conclusions.

## 5. Conclusions

This study highlighted significant distinctions in the gut microbiota of obese children in the group of children over 5 years old who were fed formula early and their normal-weight counterparts. *L. acidophilus* H-68, identified in the normal-weight children, demonstrates anti-obesity potential, which was associated with stimulating CCK secretion and producing BSH. The observed effects were multifaceted, with CCK being related to reduced food intake, while BSH inhibited liver fat deposition in mice. In addition, factors such as GLP1/PYY and TGR5 may also play a role in the anti-obesity effect of *L. acidophilus* H-68, and we plan to explore the potential mechanisms of *L. acidophilus* H-68 or other novel anti-obesity probiotics in depth from these dimensions in the future.

In all, this promising outcome promotes future research dedicated to obesity prevention in formula-fed children. Potential strategies may encompass the inclusion of *L. acidophilus* H-68 in formula or the exploration of dietary supplements for children. These initiatives will play a pivotal role in addressing pediatric obesity and fostering long-term health in formula-fed children.

## Figures and Tables

**Table 1:** Study participants’ characteristics and demographics.

Variable	OB-FF	N-FF	*p*-Values
Male/female	10/7	14/14	/
Age, years	10.27 ± 2.66	9.07 ± 2.33	0.1167
Height, m	1.48 ± 0.17	1.33 ± 0.14	0.0035
Weight, kg	59.55 ± 17.79	29.11 ± 8.24	<0.0001
BMI, kg/m^2^	26.48 ± 3.69	15.97 ± 1.63	<0.0001

OB-FF: formula-fed children with obesity, N-FF: formula-fed children with normal weight. BMI: body mass index.

**Table 2:** Primers used for the analysis of the mRNA expression levels.

Gene	Forward Primer	Reverse Primer
*SREBP-1C*	GCGCTACCGGTCTTCTATCA	TGCTGCCAAAAGACAAGGG
*FAS*	CTGCGGAAACTTCAGGAAATG	GGTTCGGAATGCTATCCAGG
*FXR*	ACATCCCCATCTCTCTGCAC	TGTGAGGGCTGCAAAGGTTT
*SHP*	CGATCCTCTTCAACCCAGATG	AGGGCTCCAAGACTTCACACA
*β-actin*	CCTAAGGCCAACCGTGAAAAG	TCTTCATGGTGCTAGGAGCCA

## Data Availability

The original contributions presented in the study are included in the article, further inquiries can be directed to the corresponding author.
